# A comparison of cyst wall curettage and en bloc excision in the treatment of aneurysmal bone cysts

**DOI:** 10.1186/1477-7819-11-109

**Published:** 2013-05-23

**Authors:** Pawel Flont, Marta Kolacinska-Flont, Kryspin Niedzielski

**Affiliations:** 1 Clinic of Orthopaedic and Traumatology, Polish Mother’s Memorial Hospital Research Institute, Rzgowska 281/289, Lodz 93-338, Poland; 2 Clinic of Internal Diseases, Asthma and Allergy, Medical University of Lodz, Kopcinskiego 22, Lodz 90-153, Poland

**Keywords:** Aneurysmal bone cyst, Children, Curettage, En bloc excision, Recurrences

## Abstract

**Background:**

The recurrence rate after aneurysmal bone cyst (ABC) treatment is quite high despite its benign nature. In ABC therapy, curettage is the treatment of choice; en bloc excision results in a lower recurrence rate, but more extensive reconstructive surgery is needed with associated morbidity. The aim of the present study was to compare the outcomes of the two treatment options.

**Methods:**

A retrospective analysis was performed on 26 patients treated for ABCs: 16 by curettage and 10 by en bloc excision. Each lesion was classified according to Enneking and patients were followed up for a mean time of 9.2 years. On follow-up, radiological examination and functional assessment (range of motion, muscle strength) were performed. Recurrence was defined as the presence of an osteolytic lesion, especially one with a tendency to grow.

**Results:**

On follow-up, the following symptoms were more prevalent in the en bloc excision group compared to the curettage group: pain (en bloc 20% versus curettage 6.25%), limb length differences (en bloc 20% versus curettage 12.5%), reduced range of motion (en bloc 20% versus curettage 6.25%) and muscle strength impairment (en bloc 50% versus curettage 31.2%); however, the differences were not statistically significant (*P* >0.05). In the curettage group, two cases of postoperative complications and two cases of recurrence were seen, while in the en bloc excision group one case of complications was noted.

**Conclusions:**

Curettage is a standard procedure in ABC management. En bloc excision is another option, albeit more technically demanding, that may be considered in recurrent lesions with extensive bone destruction or for cysts in an expendable location.

## Background

An aneurysmal bone cyst (ABC) is an expansile bone lesion that most commonly occurs in children and adolescents. In contrast to simple bone cysts, aneurysmal cysts seem to be benign neoplastic lesions with oncogene (ubiquitin C-terminal hydrolase 6 (USP6), cadherin 11 (CDH11)) and insulin growth factor activity [1-3]. Despite their benign nature, the recurrence rate of ABCs ranges from 10% to 40% [4,5]. Cyst wall curettage with or without adjuvant therapy is the treatment of choice for ABCs [6-8]. Most authors recommend curettage in the case of primary cysts and reserve excision only for recurrent cases, or for lesions located in expendable bones that can be removed surgically without the need for bone reconstruction [9-11]. Curettage is a fairly simple procedure that usually encourages relatively fast recovery, while en bloc excision result in a lower recurrence rate but more extensive reconstructive surgery is needed, with associated morbidity [5,9]. The purpose of this study was to report and compare outcomes of pediatric ABC treatment via curettage or en bloc excision.

## Methods

A retrospective analysis was performed on 26 patients referred to our clinic for ABC treatment from 1993 to 2008. The following study inclusion criteria applied: participant’s age at the time of surgery <18 years, cyst treated with one of the two surgical modalities to be studied (cyst wall curettage or en bloc excision), surgical procedure performed at the study site, and ABC confirmed by pathology and X-ray/computed tomography (CT) (Philips Brilliance iCT 256®, Cleveland, Ohio, USA). Patients with secondary ABCs confirmed by magnetic resonance imaging (MRI) (Picker Edge 1.5T® and Philips Achieva 3.0T TX®, Cleveland, Ohio, USA) and pathology, cysts treated with other methods, or cysts located in the spine or the skull were excluded from the study. In cases where X-ray/CT, location of the lesion, patient age or clinical findings were not characteristic of an ABC, an open biopsy and MRI examination were performed to exclude such other conditions as giant cell tumor, telangiectatic osteosarcoma, low-grade osteosarcoma, chondroblastoma, osteoblastoma, angioma, fibrous dysplasia, chondromyxoid fibroma, non-ossifying fibroma, unicameral bone cyst and eosinophilic granuloma [10]. Tissue obtained during the procedure was evaluated histologically, and the following inclusion criteria for ABC (as described by Lin *et al*.) were applied: presence of blood-filled spaces, membranes composed of spindle-shaped mononuclear stroma cells, lack of organization in stroma cells, osteoclast-like giant cells scattered throughout the lesion, focal concentrated aggregates of giant cells, absence of chondroid matrix and plump mononuclear cells typical of giant cell tumors [6]. Typical MRI findings were internal septation, fluid-fluid level formation and rim over the external surface of the cyst (with possible focal break) [10]. In cases where MRI/CT examination showed a solid or calcified area, an open biopsy of the suspected area was performed to exclude secondary lesions.

The demographic data of the subjects enrolled for the study is shown in Table 1. Osseous locations were as follows: the fibula in eight patients (five in the proximal and three in the distal metaphysis), the femur in six (two in the subtrochanteric area, two in the distal metaphysis, one in the lateral condyle and one in the shaft), the tibia in four (two in the distal and one in the proximal metaphysis and one in the shaft), the humerus in two (one in the shaft and one in the proximal metaphysis), the radius in two (shaft), and the scapula (lateral border), the acromial end of the clavicle, the wing of ilium and the shaft of the fourth metacarpal bone in one patient each.

**Table 1:** Demographics, radiological type and clinical outcomes by treatment group

**Procedures**	**Curettage**	**En bloc excision**	**Total**
Sample size	16	10	26
Demographics:			
Age range at diagnosis, years	2 to 18	9 to 16	2 to 18
Mean age, years	12.69	13.3	12.92
Median age, years	14	14	14
Female, %	62.5	50	57.69
Male, %	37.5	50	42.31
Radiological type:			
Central/subperiosteal lesion	9/7	9/1	18/8
Enneking’s scale I/II/III	II 9, III 7	I 1, II 3, III 6	I 1, II 12, III 13
Clinical outcomes:			
Pain, %	6.25	20	11.54
Limb length discrepancy, %	12.5	20	15.38
Decreased range of movement, %	6.25	20	11.54
Muscle strength impairment, %	31.2	50	38.46
Recurrences, n	2	0	2
Complications, n	2	1	3

Each ABC was categorized, firstly according to Enneking’s scale for benign tumors (one of latent, active or aggressive [12]), and secondly by location: central or subperiosteal [9]. The mean follow-up period was 9.2 years from the surgery (range: 3 to 19 years). Outcomes were evaluated radiographically using X-ray images of the affected limb in standard positions [13] and by functional assessments using bilateral measurements of range of motion and maximal muscle strength. The evaluation also included measures of patient-reported outcomes.

Recurrence was defined as a radiologically-confirmed osteolytic lesion, especially if the lesion displayed a tendency to grow. In this event, the patient underwent a second surgical procedure.

Range of motion (ROM) in the joint closest to the lesion was evaluated by standard goniometric assessment [14,15]. All tests were performed bilaterally for comparison. A difference in ROM of more than 20% between sides was considered clinically significant. Limb length was assessed according to AO Foundation guidelines: a 1.5 cm difference for the lower limbs and a 2-cm difference for the upper limbs were considered significant [15]. Maximum isometric muscle force was measured in standard positions in two ROM planes using a calibrated hand-held dynamometer (Advanced Force Gauge, Mecmesin®, Slinfold, West Sussex, UK) and a difference of more than 20% between sides in at least one plane was considered clinically significant [16,17]. The Pediatric Orthopedic Society of North America (POSNA) pediatric musculoskeletal functional health questionnaire was used to evaluate the patient reported outcomes [18,19]: the results were reviewed based on the assumption that the cut-off point of the patients’ wellbeing was the 25th centile.

**Figure 1:**
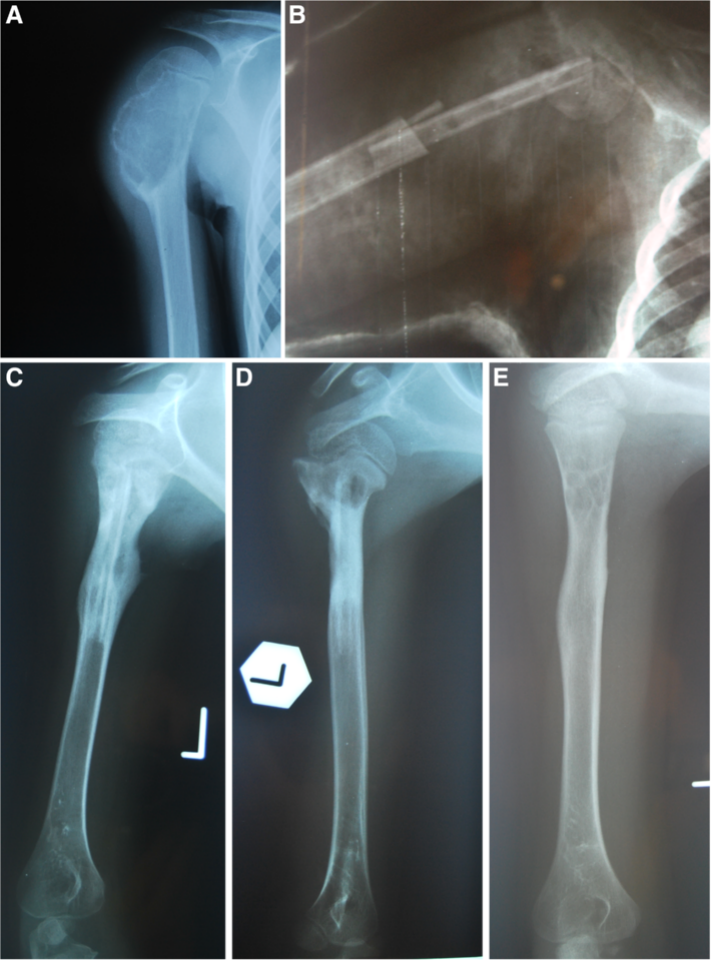
**Example of a patient with an aneurysmal bone cyst (ABC) in the humerus treated with en bloc excision of the affected bone fragment and autogenous fibular graft reconstruction.** (**A**) Preoperative X-ray of the lesion; (**B**) X-ray follow-up directly after the surgical procedure; (**C**) at 3 months postoperatively; (**D**) at 6 months postoperatively; and (**E**) final result, 3 years postoperatively. The ABC is healed with small cystic lesions.

Of the 26 patients who met the study inclusion criteria, 14 underwent cyst wall curettage, 10 en bloc excision and 2 curettage followed by en bloc excision (recurrence cases). Treatment method was selected at the discretion of the operating surgeon and was based on the site, imaging aspect and the preferences of the parent(s). In nine cases, en bloc excision was performed on the central lesion with extensive bone destruction, while in one case, in an Enneking’s stage III subperiosteal lesion, the procedure was performed in the radius shaft. In the curettage group (n = 16), seven ABC cases were subperiosteal while nine were central. In 15 of 16 cyst curettage procedures, the resection cavity was filled with bone grafts: 11 with freeze-dried bone allografts and 4 with cortical cancellous bone autografts harvested from the fibula or tibia. Each curettage was performed with the use of a bone curette and a high-speed burr. The adequacy of curettage was ascertained by direct vision and the procedure was performed until normal looking bone marrow was seen. In cases where part of the ABC was close to the epiphyseal plate or articular cartilage, only a bone curette was used under direct vision. En bloc cyst excision was performed in 12 patients, mainly in central lesions: it was used as the primary procedure in 10 patients and treatment for recurrence after curettage in 2. In six cases, resection cavities were filled with non-vascular fibular or tibial autografts (Figure 1). Bone grafts were not used in cases involving resection of expendable bones such as parts of the iliac crest, the acromial end of the clavicle, diaphysis and proximal metaphysis of the fibula. In one subperiosteal cyst where the postexcisional bone mass was large enough to provide a support function, bone grafting was not performed.

The χ^2^ independence test was used to compare various qualitative parameters in study groups and subgroups selected on the basis of different variables: the Yates correction was used for small samples. As most of the parameters analyzed in the study were not normally distributed, their means were compared using non-parametric tests: the Mann–Whitney test (for two groups) and the Kruskal-Wallis test (for several independent groups). The study was approved by our Institutional Review Board (date of issue 09.06.2009; registration number RNN/519/09/KB).

## Results

At diagnosis, symptoms were present in 17 patients (65.4%), and included pain at the affected site (n = 13), localized soft tissue edema (n = 3) and limping (n = 1). The mean time from onset of symptoms to diagnosis and treatment was 21 weeks (range 1 to 52 weeks). Only five subjects (19.2%) presented initially with pathological fractures: two with fibular cysts and individual patients with humeral, scapular and radial cysts. All pathological fractures occurred after low-energy trauma and were non-dislocated.

With regard to Enneking classifications, the majority of ABCs were stage III (benign aggressive) (n = 13), followed by stage II (benign active) (n = 12) and stage I (benign latent) (n = 1). To evaluate treatment outcomes, the patients were divided into groups by type of primary procedure. Group I were patients (n = 16) who underwent cyst curettage, group II were patients (n = 10) who underwent cyst en bloc resection. No statistically significant differences in age (z = 0.378; *P* >0.05) or sex (χ^2^ = 0.394; *P* >0.05) were found between the treatment groups. Table 1 presents the demographics, radiological type and clinical outcome data according to treatment group.

On follow-up, the following symptoms were more prevalent in the en bloc excision group compared to the curettage group: pain (en bloc 20% versus curettage 6.25%), limb length discrepancy (en bloc 20% versus curettage 12.5%), decreased ROM (en bloc 20% versus curettage 6.25%) and muscle strength impairment (en bloc 50% versus curettage 31.2%), but these differences were not statistically significant (*P* >0.05). Moreover, no intergroup differences were found in patient-reported outcomes (*P* >0.05). The POSNA survey revealed that out of the entire group of ABC cases, sport performance impairment (73.1% of the whole study) was most commonly experienced symptom, followed by decreased happiness (11.6%), increased sensation of pain (7.7%), impaired upper extremity function (3.8%) and impaired transfer and basic mobility (3.8%).

Only three patients in the whole study population (two in the curettage group and one in the en bloc resection group) developed serious postoperative complications. These are outlined below.

The first patient experienced soft tissue inflammation related to ABC in the proximal tibia metaphysis after curettage with freeze-dried bone allografts requiring reoperation, debridement of the inflammatory lesion and implantation of a gentamicin-collagen sponge. The cause of the inflammation remained undetermined as the cultures came back negative. However, the inflammation resolved itself after debridement and the graft became incorporated and consolidated completely, with full function of the limb being restored.

The second patient experienced valgus deformity (10 degrees) in the area of the distal femoral metaphysis, associated with iatrogenic epiphyseal plate damage during curettage or with direct cyst compression on the plate. The patient, a boy, was 13 years old at the time of surgery. The slight valgus deformity was noticed a year after the operation and increased to 10 degrees, measured from the mechanical axis on a weight-bearing radiograph, by the age of 16. The parents and the patient were informed of the possibility to perform an osteotomy but refused surgery.

The final patient experienced lateral instability of the knee joint after excision of the proximal part of the fibula. The patient reported lateral knee pain on exertion and a feeling of knee instability when not wearing a brace, but without functional limitation. The patient is scheduled for lateral collateral ligament reconstruction after completion of skeletal growth.

With regard to treatment complications, two cases of postoperative complications and two recurrence cases were seen in the curettage group (n = 16) whereas one case of postoperative complication was noted in the en bloc excision group.

No pathological fractures were observed in the study group in the course of treatment. The cyst relapsed in two patients, both in the curettage group. The first case, a 15-year-old patient with distal tibial metaphysis ABC experienced a recurrence about 18 months after surgery. In the second case, the relapse occurred approximately 6 months postoperatively in a 2-year-old patient with a distal fibular metaphysic cyst; the two recurrent lesions were treated by resection of the entire affected bone fragment with concurrent implantation of non-vascular fibular autograft. All cysts that recurred were classed as Enneking’s stage III: benign aggressive tumors. No local recurrences were observed at Enneking’s stage I and II.

## Discussion

Curettage remains the current standard of ABC treatment [6-8]. Some authors also report use of en bloc excision but use it only for expendable locations or in the event of multiple recurrences [5,9]. Alternative treatment via radiotherapy has been used in the past, but due to the possible induction of malignancy it is no longer employed [9,20]. Embolization [21,22] or sclerotherapy [23-25] seems a valid alternative to surgery, especially in ABC cases of the axial skeleton (spine, sacrum, pelvis) where surgery is contraindicated, especially in inaccessible lesions. Embolization may be a primary treatment in lesions with substantial soft tissue expansion in the proximity of neurovascular structures such as the spine, or in large lesions to minimize intraoperative hemorrhage, for example, in the pelvis [21,22,26]. Recent advances in ABC treatment also include curettage with an adjuvant such as phenol, hydrogen peroxide, liquid nitrogen, poly(methyl methacrylate) or argon beam, which results in a reduced recurrence rate by broadening the zone of tissue necrosis to include possible residual microscopic tumor cells [11,27-32].

The demographics of our group were not markedly different from that of previous epidemiologic studies; the age of the group in the present study is 12.9 years, versus 11.1 [33] or 13 [34] years in others. The sex distribution in our study was typical, and comparable to that presented in a previous meta-analysis; the male/female ratio of our group is 42.3%/57.7% versus 46.9%/53.1% of Shreuder *et al*.’s meta-analysis [31].

### Clinical presentation

Previous studies have shown pain (in 93% to 65% of cases) and swelling (in 15 to 18.1%) to be associated with ABCs at diagnosis. Palpable tumor and joint contracture are less frequent (8%) and the prevalence of pathological fracture varies from 3.4% to 25.9% [5,10,31]. In the present study, the frequency of pain (50%) and local edema (11.5%) were found to be lower. Such an under-representation of patients reporting pain and edema in our study may result from the fact that our cohort consisted only of children, and hence the data was partially obtained from medical histories taken mainly from parents, which may not reflect the actual condition of the patient. In our group, the time from onset of symptoms to treatment (21 weeks) is comparable with that seen in other studies (14 weeks [5], 24 weeks [10], 27 weeks [35]). A similar delay in ABC diagnosis in our patients implies that the disease stage was similar, and therefore enables the rates of recurrence and complications to be compared with those of the literature data.

### Bone growth impairment

Most authors do not discuss bone growth impairment after surgical treatment of ABCs because the study populations include children and patients with completed skeletal growth. In our study, four patients (15.5.%) developed bone growth impairment. The mean age of these subjects at the time of procedure is comparable with that of the group with normal limb development: 12.2 years and 13 years. However, in all those patients, the cysts were directly adjacent to the growth plate. Lin *et al.* noted only a 5.3% rate of growth abnormalities that cause limb shortening and axis deviation. The authors suggest an association between growth impairment and cyst recurrence and damage to the growth plate during surgery [6]. Ramírez and Stanton noted a slightly higher percentage of shortenings, 7.5% of patients (n = 3), and suggest on the basis of MRI results that damage to the growth plate caused by the cyst is directly responsible for growth impairment [35].

### Recurrences

In a retrospective study evaluating 185 patients, Campanacci *et al.* found that the time to recurrence for ABCs was 2 to 72 months [9]. Similarly, Ramírez and Stanton reported that the mean time to recurrence was 18.7 months (range: 4 to 39 months) [35]. In our study, the minimum follow-up period of 3 years seems sufficient to reliably assess recurrence.

Vergel de Dios *et al*. reported a 21.8% recurrence rate after curettage and did not note any relapses in patients with en bloc excision. A total of 90% of recurrences occurred in subjects below 20 years of age, and the mean age of the patients with treatment failure was lower than the mean age of the whole group (13.1 versus 16.1). The authors associated recurrence with younger age, but the statistical analysis did not confirm this [10]. In our study group, the mean age of patients (12.9 years) is comparable to that of the patients with recurrences in the study by Vergel de Dios *et al*. (13.1 years), but no greater frequency of treatment failures was observed. The authors also suggest that recurrences are associated with the cyst being located in long bones, and that a more favorable prognosis is associated with flat bones. They consider lesion curettage the treatment of choice for ABCs; they state en bloc excision should only be used for lesions in locations enabling affected site resection without functional limitation [10].

Ramírez and Stanton also noted a 27.5% recurrence rate after ABC treatment, as well as a halving of recurrence rate (from 40% to 21%) after the introduction of a high-speed burr. As 75% of recurrences were seen in children below 5 years of age, the authors hypothesize that ABCs occurring in this age group display a more aggressive evolution. Ramírez and Stanton also suggest that unsatisfactory treatment results may be associated with fear on the part of the surgeon of damaging the growth plate in younger patients [35]. According to Campanacci *et al*., the risk of recurrence after curettage is as high as 21%. Relapses were more common in active (25%) and aggressive (26%) lesions, but absent in cases of curettage of inactive cysts and after en bloc excision. Considering those findings, Campanacci *et al*. proposed deeper curettage with addition of phenol or cryotherapy in the treatment of more aggressive cysts. Bone excision was also recommended as the treatment of choice when the cyst is located in the proximal part of the fibula, distal part of the ulna, in ribs, pubic bone rami, and metatarsal and metacarpal bones [9]. Gibbs *et al*. reported a statistically significant correlation between the younger age of the patient, the presence of open growth plates and a higher rate of recurrences. While relapses occurred in four patients (12%) aged 3, 4, 10 and 11 years in the curettage group, no such cases occurred in patients treated with en bloc excision. As noted previously, the authors link higher risks of recurrence to more aggressive lesion types or insufficient excision of the pathological tissue in the growth plate area. On the basis of their own experience and the literature, Gibbs *et al*. hypothesized that the use of additional chemical or physical resection does not result in improved treatment outcomes [5].

Lin *et al*. reported an 18.9% recurrence rate is associated with curettage. The cysts recurred significantly more often in patients aged <12 years and in cases when lesions were directly adjacent to the growth plate or articular cartilage [6].

Similarly, the recurrence rates after curettage and en bloc excision determined by Mankin *et al*., 22% and 5%, respectively, were higher than in those found in the present study. The authors saw a higher recurrence rate in children below 10 years of age (24%), but the correlations were not statistically significant. Furthermore, the study indicated a higher recurrence rate for cysts located in the clavicle (50%) or in the distal femur (46%), but this suggestion was based only on a few cases. Out of 150 patients participating in their study, 34 were reoperated, and 11 underwent 3 surgeries. The authors recommend curettage as a treatment of choice and en bloc excision of cysts of the femur, clavicle, foot bones, scapula and fibula [11].

Schreuder *et al*. demonstrated the efficacy of additional chemical resection in ABC surgery and recorded a recurrence rate lower than in our findings (3.7%). The burred surface was covered with sprayed liquid nitrogen several times during the procedure, which allowed the margin of bone excision to be extended without being weakened and forestalling the need for reconstructive surgery. No postoperative wound healing disorders occurred, nor any postoperative fractures [31].

A statistically significant correlation was seen between recurrence and use of adjuvant (*P* = 0.044) in a study by Cummings *et al*. that evaluated the effectiveness of curettage with or without the use of an argon beam [30]. The authors did not observe any relapses in the group treated with an adjuvant argon beam and no perioperative or postoperative complications were seen. Another evaluation of curettage surgery with an argon beam, by Steffner *et al*., also reported a statistically significant correlation between the risk of the recurrence and use of the argon beam [29].

Where surgery is contradicted due to a difficult surgical approach, embolization [21,22] or percutaneous sclerotherapy [23-25] can be an optional treatment: another indication for embolization being a reduction in operative blood loss that facilitates curettage, especially in the pelvis or spine [26]. Both techniques can be used in cases of postsurgical recurrence. Embolization is usually performed with use of polyvinyl alcohol, *N*-2-butyl cyanoacrylate or Ethibloc® [36]. De Cristofaro *et al*. described the treatment of 14 patients with ABC where polyvinyl alcohol was used. Although embolization was not possible in 5 of these patients due to lack of a recognizable artery or common vascular supply to the lesion and spinal column, the authors obtained almost complete ossification in 11 cases, recurrences with pathological fracture in 2 cases and an unchanged cyst in 1 case, after a mean number of 2.3 procedures per patient. No complications were reported [21].

The most encouraging results can be seen in a paper by Rossi *et al*., who indicated that arterial embolization with cyanoacrylate may be the treatment of choice for ABC because it is less invasive, cheaper and simpler than surgery and is easily repeatable. The treatment was found to be effective in 32 patients (94%) and complications were noticed only in 3 procedures, which were cases of skin necrosis and transient paresis [22]. Although recent studies show this treatment to have good results, pulmonary embolism or ischemia of vital structures not related to the tumor can occur [37].

Another new procedure that is both simple and minimally invasive is the aforementioned percutaneous sclerotherapy using a thrombogenic substance, which induces an inflammatory reaction to a foreign body but lacks osteogenic properties. The use of one such substance in ABC management, Ethibloc®, was first reported by Adamsbaum *et al.*, who achieved satisfactory healing in two cases [38]. Other studies show that percutaneous embolization may be considered a reliable alternative to surgery but, due to possible complications, this therapy is recommended only in deep located ABCs without marked venous drainage. Guiband *et al*. treated 14 patients with ABCs with total lesion healing in 13 cases (87%) and partial healing in 2 cases (13%), without recurrences, the complications being 1 case of aseptic osteitis and a small pulmonary infarct [23]. Garg *et al.*, who assessed the outcomes of CT-guided percutaneous injection of Ethibloc® into ABCs in ten patients, obtained total resolution of the lesion in seven patients and partial response in three, with the only complication being an aseptic abscess [24]. However, Topouchian *et al*. reported a high rate of local and general complications after Ethibloc® injection including one pulmonary embolism and four aseptic fistulizations; the authors abandoned fibrosing agent injections in the treatment of ABC [39]. In a comparison of sclerotherapy with curettage in ABC treatment by Varshney *et al*., it was reported that repetitive sclerotherapy using polidocanol is a minimally invasive method that is safer than curettage, although similar recurrence rates are noted for the two treatment methods [25].

In the study group used in the present work, more radical treatment methods than those presented in the previous studies were used. En bloc excisions of bone fragments were performed in as much as 40% of patients, especially in the case of an Enneking’s III central lesion or expendable bones, which could be associated with more frequent postoperative complications. In the group of en bloc excision, only one complication occurred, versus two complications in the curettage group. In fact, it could not be demonstrated that bone fragment excision was associated with more frequent postoperative complications. Unfortunately, due to the small size of the sample, this remains only a hypothesis. It should also be noted that excision of a bone fragment and its replacement with bone graft is technically more difficult, more time consuming and often requires autograft bone harvesting, therefore it is not a standard treatment.

Pain and ROM limitation on follow-up were more common in the group where en bloc excision of bone fragments was performed, but no statistically significant differences between the procedures were found. All issues reported by our patients were mild in nature and occurred in rather non-specific situations, and although they did not interfere with any of our patient’s daily activities, they might impair athletic performance. Moreover, reduced ROM in this group applied usually to only one plane. Furthermore, no significant intergroup difference was found in muscle strength assessment. However, weakening of isometric muscle force in at least one range was observed in as much as half of the patients in the en bloc excision group: four patients in one plane, and one in two planes. This may result from soft tissue damage during an extensive procedure, from a pathological fracture or from prolonged postoperative immobilization in comparison with the curettage group. Muscle force reduction in eight patients applied only to one ROM plane, four from the curettage group and four from the en bloc excision group. In the remaining two patients, one in each treatment arm, the limitation applied to two ROM planes, and exceeded 50% when compared to the healthy side. Such disproportion seriously affects the physical activity of the patient.

The weakness of our study is an insufficient number of patients to draw statistically significant conclusions. For this reason, we limited this paper to cohort description and to forming hypotheses that should be investigated on larger patient samples. However, taking into account the low annual incidence of primary ABC (0.14 per 100,000 individuals), building up a more numerous study cohort would be a challenge [34].

## Conclusions

Curettage is a standard procedure in ABC treatment. En bloc excision is another option, more technically demanding, that may be considered in recurrent lesions with extensive bone destruction or for cysts in an expendable location.

## Abbreviations

ABC: Aneurysmal bone cyst; AO Foundation: Arbeitsgemeinschaft für Osteosynthesefragen; CDH11: Cadherin 11; POSNA: Pediatric Orthopedic Society of North America; ROM: Range of motion; USP6: Ubiquitin C-terminal hydrolase 6.

## Competing interests

The project is supported by a grant from KBN (Polish funding agency) no. 5866/B/P01/2010/38. We certify that there is no conflict of interest with any financial organization regarding the material discussed in the manuscript.

## Authors’ contributions

PF: preparing the material, describing the data, carrying out the clinical and radiological assessment, translating the text (50%). KN: coordinating and supervising the study and manuscript preparation, preparing the photo documentation, preparing the manuscript (40%). MK-F: preparing the manuscript, translating the text (10%). All authors read and approved the final manuscript.
